# Investigation of Iron and Zinc Concentrations in Human Milk in Correlation to Maternal Factors: An Observational Pilot Study in Poland

**DOI:** 10.3390/nu13020303

**Published:** 2021-01-21

**Authors:** Agnieszka Bzikowska-Jura, Piotr Sobieraj, Magdalena Michalska-Kacymirow, Aleksandra Wesołowska

**Affiliations:** 1 Department of Clinical Dietetics, Faculty of Health Sciences, Medical University of Warsaw, E Ciolka Str. 27, 01-445 Warsaw, Poland; 2 Department of Internal Medicine, Hypertension and Vascular Diseases, Faculty of Medicine, Medical University of Warsaw, Banacha Str. 1a, 02-097 Warsaw, Poland; piotr.sobieraj@wum.edu.pl; 3 Faculty of Chemistry, Biological and Chemical Research Centre, University of Warsaw, Żwirki i Wigury Str. 101, 02-089 Warsaw, Poland; m.kacymirow@cnbc.uw.edu.pl; 4 Laboratory of Human Milk and Lactation Research, Regional Human Milk Bank in Holy Family Hospital, Faculty of Health Science, Department of Medical Biology, Medical University of Warsaw, Litewska Str. 14/16, 00-575 Warsaw, Poland; aleksandra.wesolowska@wum.edu.pl

**Keywords:** breastfeeding, human milk composition, iron, zinc, maternal diet, nutritional status, nutrition requirements, nutritional deficiencies

## Abstract

The aim of this study was to evaluate iron and zinc concentrations in the mature human milk (HM) and to investigate the relationship between these concentrations and maternal factors. HM samples were collected between 4–6 weeks postpartum from 32 healthy, exclusively breastfeeding mothers. The assessment of dietary intake during breastfeeding was based on a food frequency questionnaire and three-day dietary records. Nutritional status of participants was assessed with body mass index and body composition analysis, measured with bioelectrical impedance. HM intake was assessed with infants’ weighting, whereas iron and zinc contents in HM were determined by inductively coupled plasma mass spectrometer. The median intake of HM was 492.5 mL (466–528.5) and the concentrations of HM iron and zinc were 0.33 mg/L (0.26–0.46) and 2.12 mg/L (1.97–2.45), respectively. Maternal total zinc and iron intake (diet + supplementation) was positively correlated with their concentrations in HM. Consumption frequency of meat, vegetables and legumes was revealed to be a significant factor influencing zinc concentration in HM. Regarding iron, it was the consumption frequency of meat, fish and seafood, vegetables and legumes, nuts and seeds. The intake of iron from HM was low, and after assuming a mean fractional iron absorption, it was only 0.038 mg/d. Our results show that maternal diet influences iron and zinc content in HM, suggesting that adequate intake of food rich in investigated minerals may be a positive factor for their concentrations in HM.

## 1. Introduction

The World Health Organization (WHO) recommends breastfeeding as the exclusive method of nutrition for newborns and infants at least up to six months of age and then, continuation along with complementary foods for 24 months or longer. Breast-fed infants have been shown to be better protected than formula-fed infants against many diseases, e.g., inflammatory bowel disease, necrotizing enterocolitis, lower respiratory tract diseases, type 1 and type 2 diabetes mellitus, multiple cardiovascular diseases (hypertension, arteriosclerosis), obesity, atopic dermatitis and asthma [1]. For exclusively breastfed infants, human milk (HM) must be nutritionally adequate and provide all trace elements needed for optimal growth and development. While trace minerals concentration is relatively low in HM, they play an essential role in many physiological processes [2].

Iron is a part of hemoglobin and a structural component of a variety of enzymes crucial for a range of human metabolic processes. Infants are particularly susceptible to the consequences of iron deficiency due to rapid growth and brain development [3]. Newborn needs for iron are met through the utilization of hepatic reserves accumulated mainly during the third trimester of pregnancy [4]. A newborn’s iron stores can be increased by about 30–35 mg through delayed clamping (e.g., two minutes after birth) of the umbilical cord, with a calculated difference in serum ferritin concentration of 4 μg/L, resulting from the high hemoglobin content of fetal blood and from placental sources [5]. The estimated daily physiologic requirement for iron in infants is 0.7 mg for growth and 0.2 mg to replace basal loses [6]. Chaparro et al. [7] noted that, due to the redistribution of iron from hemoglobin to iron stores, in term, healthy and normal birth weight infants had sufficient iron for the formation of hemoglobin and myoglobin concomitant with growth until about six months of age, in fully breastfed infants. Extra iron requirements during the first six months of life should be provided by HM intake. In HM, iron is bound primarily to low-molecular-weight peptides, fat globules, and lactoferrin, with the mean iron saturation of lactoferrin varying from 2.2% to 12% [8]. The iron content in HM reaches a maximum in colostrum and subsequently decline through the first year of lactation, with reported median values ranging from 0.04–1.92 mg/L (mean value—0.3 mg/L) [4,8,9,10]. According to Polish Nutritional Standards [11], the adequate dietary intake (AI) of iron is 0.3 mg/d for 0–6 months of age.

Zinc has a wide array of vital physiological functions. It plays a catalytic role in each of the six classes of enzymes. As a structural component of transcription factors, zinc also has a key role in the regulation of gene expression and is involved in signal transduction and neuronal transmission [12]. Zinc deficiency in infants results in stunted growth and compromised immune function, with increased morbidity and mortality from respiratory infections and diarrhea [4,13]. The European Food Safety Authority [14] states, that the estimated zinc requirements for infant growth are 120 and 140 µg/kg body weight per day for female and male infants, respectively, for the first three months of life. For infants up to six months of age it was assumed that the inter-individual variation of zinc requirements is 12.5% and is the same for breast-fed and formula-fed infants. Polish experts indicated that, in the first six months of life, AI for zinc is 2 mg/d [11]. The concentration of zinc in HM decreases sharply from colostrum to transitional milk, followed by a gradual decline throughout lactation [4]. It is reported that mean daily zinc transfer to the infant via HM is about 4 mg in colostrum, 1.75 mg at one month and 0.7 mg at six months postpartum [15].

Deficiencies of iron and zinc often coexist and are public health concerns during pregnancy and infancy, especially in developing countries. It is well documented that, adequate maternal nutrition, before, during and after pregnancy is crucial for the health of both, mother and child. Poor nutrition in pregnancy may result in inappropriate nutrients portioning between the mother and fetus, which can be detrimental for both. Zinc deficiency is predicted to be responsible for 1% of all deaths globally and 4.4% of deaths in children aged from six months to five years. The WHO prioritized minimizing zinc deficiency in developing countries as part of the Millennium Development Goals to fight against poverty and hunger. What is more, the results of some studies [16] suggest that gestational zinc deficiency may reduce the learning ability, attention and memory in offspring, since the hippocampus has a high zinc concentration. In turn, iron deficiency during pregnancy may lead to severe consequences, including an impaired immune-inflammatory system, premature birth and fetal death [17]. The results of some studies [18,19] have shown that even exclusively breasted infants may be at risk of zinc and iron deficiencies. Additionally, some authors [20,21] have reported a higher prevalence of these micronutrient deficiencies among breastfed infants compared to infants fed by formula or mixed feeding (HM + formula). While the proteins transporting these minerals across the mammary gland epithelium were previously characterized in cells and in animal studies, still little is known about the mechanisms and factors regulating the concentrations of these elements in HM [22].

The influence of maternal factors on the quality and quantity of HM is a frequent topic of discussion and research. However, the impact of maternal diet on iron and zinc concentrations does not have well defined effects. Some previous reports [23,24] noted that the levels of iron and zinc in HM were associated with maternal factors (e.g., dietary intake or micronutrients supplementation), whereas others suggested that zinc concentration in HM is refractory to maternal status [25], dietary intake [24,26] and supplementation [27]. In Poland, previous research concerning the content of iron and zinc in HM were conducted six years ago [28]. No detailed evaluations of the effect of maternal factors, like age, diet and nutritional status on the content of indicated minerals in HM are available. Therefore, the main aim of this study was to investigate the concentrations of iron and zinc in human milk in correlation with maternal factors (e.g., age, current and habitual dietary intake, nutritional status assessed with BMI and body composition).

## 2. Materials and Methods

### 2.1. Participants and Study Design

**Figure 1:**
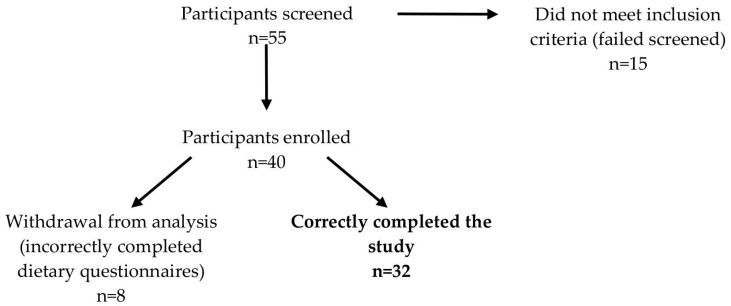
Study participant recruitment flow chart.

This was an observational pilot study with participating Polish women providing milk samples at 4–6 six weeks postpartum. Fifty-five mothers from the Department of Obstetrics and Gynecology in Holy Family Hospital and Institute of Mother and Child in Warsaw, Poland, were screened for this study. Fifteen women did not meet the recruitment criteria. A total of forty participants who fulfilled the inclusion criteria (age ≥ 18 years, singleton and full-term pregnancy, no gestational diseases, no smoking during or after pregnancy, sufficient milk supply, infants’ birth weight ≥ 2500 g and exclusive breastfeeding) were enrolled in the study. Due to incorrectly completed questionnaires, eight women were withdrawn from further analysis (Figure 1).

The study session was divided into three parts. In the first one, we asked participants about basic information, such as: age, educational level, economic status and pre-pregnancy anthropometric parameters (weight and height) and total weight gain during pregnancy. We also collected data concerning the number and volume of daily feedings. As suggested by the Wollridge et al. [29], to measure the volume of daily milk intake, all mothers were asked for the weights of their infants before and after each feeding, using a balance scale accurate to ±1 g. On this basis, we calculated daily energy and nutrients intake from HM by multiplying the nutrient or energy content and mean daily milk intake. For example, if an infant ingested 412 mL of milk with a fat content of 3.2 g/100 mL, the nutrient intake on that day was 13.18 g. Next, we asked for completed nutritional questionnaires and performed anthropometric measurements and body composition analysis. 

### 2.2. Maternal Dietary Intake

The assessment of maternal diet was based on two methods—current and habitual intake. For assessing current intake, self-administrated food dietary records on three days before milk sampling were obtained. Women were asked to note each food, fluid and dietary supplement they had consumed. No dietary recommendations were given before the study. To verify the declared food portions, we used “Album of Photographs of Food Products and Dishes” from the National Food and Nutrition Institute [30]. Additionally, women were also informed that ingredients of mixed dishes and the amount consumed should also be weighted to determine the weight of the actual ingredients consumed by the participant. Then, self-administrated semistructured food frequency questionnaire (FFQ) was used to assess the consumption frequency of selected food items over the three months prior to study. Food products in the FFQ were combined into 10 comprehensive food groups: grained based products, meat, fish and seafood, milk and milk products, eggs, vegetables and legumes, fruits, nuts and seeds, vegetable oils, sweets and snacks. The response options were arranged in five categories, from ‘never’, ‘less than once a week’, ‘once or twice a week’, ‘more than twice a week but not every day’, to ‘every day’. The FFQ was adopted from guidelines developed by the WHO [31]. Clinical dietitian checked both questionnaires (3-day dietary records and FFQ) to ensure that they were completed properly. At this stage, eight out of forty participants were excluded from further analysis, because they did not complete the FFQ questionnaire and/or dietary records was filled inaccurately. Total energy and nutrients intakes were estimated using Dieta 6.0 nutritional software (National Food and Nutrition Institute, Warsaw, Poland). Data about the content of iron and zinc in different dietary supplements was taken from the manufacturer’s websites. The estimated average requirements (EARs) were compared with Polish Nutritional Standards for breastfeeding women [11].

### 2.3. Anthropometric Measurements and Body Composition Analysis

Body weight and height were measured using a Seca 799 measurement station and column scales (±0.1 kg/cm; Seca, Chino, CA, USA). The body mass index (BMI) was calculated as the ratio between the body weight and the height squared (kg/m^2^) and interpreted in accordance with WHO classification [32]. To assess the nutritional status of participants, we made body composition analysis based on bioelectrical bioimpedance using the Maltron BioScan 920-II (Maltron Bioscan, Rayleigh, United Kingdom). All measurements were performed with a validated protocol [33], as discussed in our previous studies [34,35].

### 2.4. Milk Sample Collection and Composition Analysis

HM samples were collected one day before body composition analysis and on the third day of three-day dietary records. All women were asked to provide four pre and post-feeding HM samples, in the morning (6.00–12.00), in the afternoon (12.00–18.00), in the evening (18.00–24.00) and at night (24.00–6.00). At each time point, 5–10 mL of pre- and post-feed samples were obtained manually from the breast(s) that the infant fed from. The contents of energy, carbohydrates, fat and protein in HM were determined using the MIRIS human milk analyzer (HMA) (Miris, Uppsala, Sweden) with a validated protocol. The analysis was based on semisolid mid-infrared (MIR) transmission, detecting OH stretch in lactose on the 9.61 µm wavelength, C=O for fat on the 5.73 µm wavelength and C-H for fat on the 3.48 µm wavelength, and CO-N stretch in protein on the 6.46 µm wavelength. The MIRIS HMA provided a calculation of energy using conversion factors of 4.0, 9.25, 4.4 kcal per 100 mL for lactose, fat and protein, respectively. Total protein refers to total nitrogen × 6.25, and true protein is the total protein minus 24% for nonprotein nitrogen. Total protein, as reported by the Miris analyzer, was converted to bioavailable protein (true protein) for the data analysis using the following equation: total protein (grams) × 0.825. From each pool, three samples (~12 mL in total) were taken to analyze the nutritional value, and for the final result, we used the average of three measurements. The detailed information about HM milk collection and analysis of energy and macronutrients content was described in our previous studies [34,35].

For samples’ digestion, a microwave-assisted system Ethos Up (Milestone S.r.l, Sorisole, Italy) was used. Iron and zinc contents were determined by an inductively coupled plasma mass spectrometer (ICP-MS) (NexION 300D, Perkin Elmer SCIEX, Waltham, MA, USA). 1 mL of 65% HNO_3_ and 0.5 mL 30% H_2_O_2_ was added to approximately 0.5 g of milk sample. Then, a mineralization procedure was performed in Teflon vessels in a closed system with microwave assistance. Table 1 shows the conditions under which mineralization was carried out.

Table 2 presents selected measurement parameters optimized for the determination of chosen elements using the ICP-MS method.

In order to determine the analytical capabilities of ICP-MS and selection of the isotope, the content of chosen elements in the reference material ERM-BD150 was determined. The use of the optimized mineralization procedure (Table 1) and optimal measurement conditions (Table 2) allowed us to achieve the limits of quantification (LOQ) and the limits of detection (LOD) of the procedure. For iron, the LOQ and LOD were 1.40 mg/L and 0.95 mg/L, respectively, for zinc it was 1.6 mg/L (LOQ) and 1.49 mg/L (LOD).

### 2.5. Statistical Analysis

Discrete variables were presented as a number and percentage. Continuous variables were presented using the mean followed by standard deviation and median with the interquartile range. The normality of the distribution of continuous variables were verified using the Shapiro-Wilk test. The relationship between maternal factors and HM iron and zinc concentrations was evaluated using the Pearson and Spearman correlation coefficients (depending on distribution of the variables). The results of statistical testing were considered significant when the *p*-value was <0.05. Multivariable linear regression models were used to assess the effect of mother’s age, actual body mass, body fat mass, percentage of energy delivered from macronutrients (protein, fat, carbohydrates), consumption frequency of selected food groups (meat, fish and seafood, vegetables and legumes nuts and seeds), dietary, supplemented and total iron intake were used as explanatory variables for the prediction of HM iron concentration in HM and infants’ iron intake. Due to the fact that the concentration of zinc in 15 HM samples was below the lower limit of quantification, models for zinc concentration were developed similarly; however, logistic instead of linear regression was used. The dependent variable in the logistic regression models was coded as dichotomous using the value of the 90th percentile of zinc concentration in HM as a threshold. In 15 HM samples with the HM zinc concentration below the lower limit of quantification, infants’ zinc intake was estimated by multiplying the lower limit of quantification by the volume of HM. However, zinc intake was much lower than EAR (2 mg per day). Moreover, as will be shown below, in the whole study population, HM zinc infants’ intake was much lower than EAR. The highest zinc infants’ intake was 1.44 mg, representing 72% of the EAR. For this reason, our paper reports the infants’ zinc intake for a group with a quantifiable HM zinc concentration. For the above-mentioned reasons, it would not be clinically meaningful to further analyze the infants’ zinc intake, including modeling. For the linear and logistic regression models, Akaike Information Criterion (AIC) was used for simplification of the evaluated models. Results of multivariable logistic regression were presented as the odds ratio (OR) with a 95% confidence interval (CI) for one-unit increments of each variable. All computations were performed in R 3.6.0 (R Foundation for Statistical Computing, Vienna, Austria), an environment for statistical programming. Standard, ‘psych’, ‘glmulti’, ‘rms’, ‘oddsratio’ ‘ggplot2’, and ‘cowplot’ packages were used.

## 3. Results

### 3.1. Characteristics of Participants

Table 3 presents characteristics of the study population. Mean age of participants was 33.8 ± 4.6 years. All women had a university education and declared a high or very high socioeconomic status. Most of them (*n* = 24, 75%) were primiparous and gave birth to a baby boy (*n* = 20, 63%). Nutritional status assessed with BMI indicated that most of women (*n* = 24, 75%) had normal body mass and 25% (*n* = 8) were overweight. Mean fat mass content in the women’s bodies was 29.1 ± 9.0%.

### 3.2. Human Milk Composition and Estimated Nutrients Intakes 

The nutrients profile of HM is presented in Table 4. The median concentration of iron in HM was 0.33 (0.26–0.46) mg/L, while infants’ daily iron intake from HM was 0.16 (0.12–0.24) mg. The samples of HM from only four (12.5%) women had a total iron amount higher than daily infant demand. The correlation between HM iron concentration and daily intake of iron was strong.

In 17 samples (53%) of HM with quantifiable HM zinc concentration, the median zinc concentration was 2.12 (1.97–2.45) mg/L. Considering the same samples, mean zinc infants’ intake was 1.08 ± 0.21 mg/d (median 1.1 mg with interquartile range 1.03–1.2 mg). The highest zinc infants’ intake was 1.44 mg/d. We found correlation between iron and zinc concentrations in HM.

### 3.3. Correlation between Maternal Diet and Iron and Zinc Concentration in HM

Table 5 provides the results of the maternal nutritional value of diet and the correlation between iron and zinc concentrations in HM. The mean maternal energy intake was 1830.6 ± 485.1, whereas according to Polish Nutritional Standards [11] EAR for breastfeeding women is about 2455 kcal/d. The mean total intake (diet + supplementation) of iron and zinc was 11.2 ± 2.8 and 9.9 ± 1.8, respectively, whereas recommended dietary allowance for breastfeeding women are 10 mg and 12 mg, respectively. Almost half of the participants (*n* = 15, 47%) declared the use of dietary supplements containing iron, while only five women (16%) had supplemented zinc. Interestingly, all women who supplemented zinc supplemented iron as well.

No correlation was found between maternal dietary or supplemented iron with iron concentration in HM. What is more, no correlation was also found between total iron intake (diet + supplementation) and iron concentration in HM.

Similarly, no correlations were found between maternal diet, supplemented or total iron intake with estimated infants’ dietary intake of this mineral. 

There was no correlation between women’s dietary zinc intake and zinc concentration in HM. However, supplemented zinc and total zinc intake correlated with the zinc concentration in HM.

**Figure 2:**
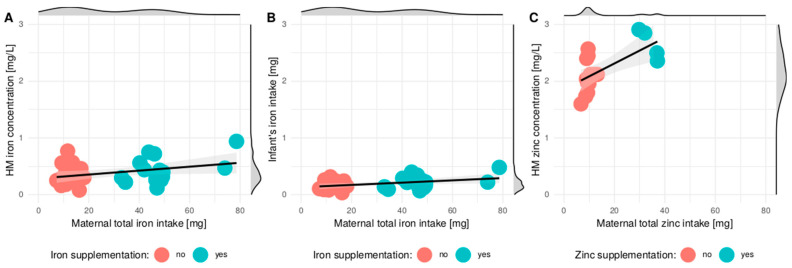
Correlation between total (dietary + supplemented) iron (**A**,**B**) or zinc delivery (**C**) and HM iron concentration (**A**), infant’s iron intake (**B**) and HM zinc concentration (**C**) according to the supplementation of iron or zinc. Density of HM iron/zinc concentration, infant’s iron intake, total iron intake and total zinc intake is presented at marginals of the plot. Linear regression lines with 95% confidence intervals were added to the plots.

Figure 2 presents the relationship between total (dietary + supplemented) iron or zinc delivery and HM iron concentration, infant’s iron intake and HM zinc concentration. 

Table 6 summarizes the results concerning data from FFQ. We did not observe any statistically significant correlation between the frequency of food consumption and zinc concentration in HM; however, the consumption frequency of eggs, vegetables and legumes was positively correlated with HM iron content. 

### 3.4. Multivariable Regression Models Evaluating the Relationship between Maternal Factors and Mineral HM Content

Several multivariable models evaluating the impact of selected factors on iron concentration in HM and infants’ iron intake were considered. Mother’s age, actual body mass, body fat mass, percentage of energy delivered from macronutrients (protein, fat, and carbohydrates), dietary, supplemented and total iron intakes were used as explanatory variables for prediction of HM iron concentration in HM (Model A) and infants’ iron intake (Model B). Both of these models, using the lowest AIC, were simplified to univariable models, implicating supplemented iron and total iron intake as significant factors, respectively. 

For models C and D mother’s age, actual body mass, body fat concentration, consumption frequency of selected food groups (meat, fish and seafood, vegetables and legumes nuts and seeds), dietary, supplemented and total iron intakes, were considered explanatory variables. The iron concentration in the HM (Model C) and infants’ iron intake (Model D) were used as dependent variables. 

On the basis of lowest AIC, the models were simplified, as presented in Table 7. Consumption frequency of meat, fish and seafood, vegetables and legumes, nuts and seeds and maternal total iron intake were identified as significant factors influencing iron concentration in HM and infants’ iron intake. 

Multivariable logistic regression models were also considered to evaluate the relationship between maternal factors and zinc concentration in HM (Models E and F). HM zinc concentration higher than 90th percentile (2.495 mg/L) was used as dependent variable. In only four samples of HM, the zinc concentration was higher than the 90th percentile. 

Mother’s age, actual body mass, body fat content, percentage of energy delivered from all macronutrients (protein, fat, and carbohydrates), dietary, supplemented and total zinc intakes were used as explanatory variables for the prediction of zinc concentration in HM (Model E). Model F included mother’s age, actual body mass, body fat content, consumption frequency of selected food groups (meat, fish and seafood, vegetables and legumes, nuts and seeds) dietary, supplemented and total zinc intake were considered as explanatory variables. 

On the basis of the lowest AIC, the final Models E and F were chosen and presented in Table 8. In Model E, body fat mass, percentage of energy delivered from proteins and total zinc intake were revealed to be significant factors influencing zinc concentration in HM. According to Model F, consumption frequency of meat, vegetables and legumes and total zinc intake affected HM zinc concentration.

## 4. Discussion

This was the first report determining the concentration of iron and zinc in HM along with assessment of nutritional status and nutrient intake of Polish women. Performing multivariable regression analysis, we reported that maternal total zinc and iron intake (diet + supplementation) positively influenced their concentrations in HM. We also noted that the consumption frequency of meat, vegetables and legumes were revealed to be significant factors affecting zinc concentration in HM. Regarding iron, it was the consumption frequency of meat, fish and seafood, vegetables and legumes, nuts and seeds. Simultaneously, we observed a strong correlation between HM iron and infant’s daily iron intake from HM. What is more, we found correlation between iron and zinc concentrations in HM. The intake of iron from HM was low, and after assuming a mean fractional iron absorption, it was only 0.038 mg/d.

In our study, the concentration of HM iron (0.33 mg/L) was higher compared to mature milk from Swedish (0.29 mg/L [34]), Honduran (0.21 mg/L [36]), Indonesian (0.26 mg/L [37]), Thai (0.19 mg/L [38]), and lower than Vietnamese (0.59 mg/L [39]) and Iranian (0.89 mg/L [24]) mothers. Regarding zinc, in our study its median concentration (2.12 mg/L) was similar or slightly higher than that reported in other countries [37,38,39,40]. Some of the divergences between the findings for HM iron and zinc concentrations may be related to the fact that iron content in HM was reported to be higher at the nighttime feeding and in hindmilk samples [27]. In turn, Doneray et al. [41] reported that zinc concentration in hindmilk was approximately 2 mg/L lower than in foremilk (3.36 mg/L vs. 5.75 mg/L). Several studies have also mentioned that some differences may be explained by geographical and nutritional influences [23,42]. Therefore, to minimize possible circadian influences on HM composition, we performed milk analysis based on 24 h collection. Each woman took a sample of mature milk four times a day, but eventually all samples from a given women (from four time periods: 6.00–12.00; 12.00–18.00; 18.00–24.00, and 24.00–6.00) were mixed and delivered for further analysis in one container. We also confirmed a systemic change between fore and hindmilk samples. The term foremilk refers to the milk produced at the beginning of the feeding and hindmilk refers to milk at the end of the feeding. 

The concentration of iron in HM is considered to be low, in relation to serum iron, since the HM iron concentration is 20%–30% of serum iron [39]. Most previous studies [39,43,44] did not find any correlation between maternal iron status and HM iron content, whereas one study showed higher HM iron concentration in severely anemic mothers (Hb < 8 mg/dl) [45]. The mechanism underlying this elevation is unclear, however some authors have discussed the possibility of preferential binding of certain nutrients (including iron) by the mammary gland [46]. Fransson et al. [45] suggested that an alternative explanation of these findings may be ‘leakage’ of iron and lactoferrin into the mammary gland due to subclinical mastitis. Concerning the impact of maternal iron supplementation on its HM concentration, Mello-Neto et al. [47] found no difference in HM iron concentration in women who took daily supplements during pregnancy and lactation vs. those who did not. Contrary to these findings, a Korean study [23] that aimed to evaluate zinc, copper and iron concentrations in transitory HM and investigated the association with maternal factors, showed that only iron was significantly associated with some of the general characteristic of the examined participants. A higher concentration of iron was detected in the milk samples that were obtained from mothers who gained less weight during pregnancy, who did not consume alcoholic beverages during pregnancy and breastfeeding and who took iron supplements. In Korea, iron supplementation is recommended to minimize the risk of anemia, caused by iron deficiency, supporting the assumption that long-term iron supplementation during pregnancy and lactation influences the HM iron concentration. Additionally, Choi et al. [23] observed that iron concentration in HM was positively associated with a mother’s dietary intake of fat, cholesterol, and magnesium, which may be due to the consumption of meat and meat products. In our study, in the multivariable linear regression analysis, the consumption frequency of food rich in iron (meat, fish and seafood, vegetables and legumes, nuts and seeds) and maternal total iron intake (diet + supplementation) were identified as significant factors influencing iron concentration in HM. These results suggest that maternal iron supplementation may increase its concentration in HM. However, there are also some detrimental aspects of iron supplementation, e.g., inhibition the absorption of other micronutrients, including zinc. It has been previously reported [48] that women receiving iron supplements can have zinc malabsorption. Nonetheless, in the present study, similarly to Korean [23], iron supplementation did not appear to influence the level of zinc in HM. Moreover, in our samples, we found a positive correlation between iron and zinc concentrations in HM. 

In industrialized and developing countries, milk intakes average approximately 550 to 650 mL/d in the first 1 to 2 months [49], whereas, in our population, it was only 492.5 mL/d. The iron intake of infants from HM, reported in our study (calculated as the product of HM volume consumed in mL/day and the HM iron concentration mg/L) was 0.16 mg/d. Iron needed to recover endogenous losses through the gastrointestinal tract (0.17 mg/d) and skin (0.08 mg/d) has been estimated to be approximately 0.25 mg/d. Iron at one year of life as hemoglobin (270 mg), myoglobin and enzymes (54 mg), and storage (53 mg/d) amounts to 109 mg above the amount present at birth (268 mg). Using this factorial approach, the total iron requirement during infancy is ~0.55 mg/d [50,51]. Assuming a mean fractional iron absorption rate of 0.20 [48], in our study, the amount of iron available for absorption from HM would only amount to 0.032 mg/d, indicating an increasing gap between the measured and recommended dietary intake of iron. However, despite the low intakes of HM iron, none of the infants presented symptoms of anemia. It may be caused by the fact that the newborn infant is endowed with iron stores and a high concentration of hemoglobin. However, between four and six months of age, there is an increased dependence on dietary iron. Dietary iron provides ~30% of the requirement for hemoglobin iron turnover, compared to 5% in adults [51]. Therefore, it appears that breastfed infants who do not receive additional iron from supplements or complementary foods are at risk of becoming iron-deficient from the fourth month of life. 

The zinc intake of infants, reported in 17 cases (milk samples with quantifiable zinc concentration), was 1.1 mg/d. With the assumptions of urinary and sweat zinc losses (20 µg/kg per day), endogenous fecal losses (50 µg/kg per day) and zinc required for new tissue accretion (20 µg/g weight gain or 30 µg/g lean tissue weight gain), the total requirement of zinc for infants would be 0.94 and 0.86 at 1 and 3 months, respectively. Considering that a mean factional zinc absorption from HM was 0.55 [50], in the present study, the amount of zinc available from HM would only amount to 0.6 mg/d. Our results are consistent with Samuel et al. [52], who reported that zinc intake from HM at 1, 3 and 6 months was 0.88, 0.48, 0.37 mg/d, respectively. They also indicated that, despite the low intake of zinc from HM, the serum levels of the infants appeared to be in the normal range. It may be that the requirement for zinc is partially offset by mobilization of hepatic zinc bound to metallothionein in the first months of life [52]. 

Contrary to iron, which has a concentration in HM a fraction of those in serum, HM zinc content is approximately one to two times higher than in serum in healthy breastfeeding women. Thus, it is more likely that maternal factors influence the HM concentration if the lactating women are zinc deficient [39]. In our study, the majority of participants (63%, *n* = 20) did not reach the EAR value for zinc (10.4 mg/d). These results are consistent with previous studies that indicated that maternal dietary zinc intake was nearly or below the recommended values [38,39,40]. Data from several developing countries where zinc intake was presumed to be low (e.g., Bangladesh [53], Egypt [54], and Nigeria [55]), showed that HM zinc concentrations were relatively lower than those of well-nourished American women [56]. However, Sian et al. [57] observed that Chinese lactating women at two months post-partum, were able to secrete 2 mg of zinc per day into their milk, in the presence of low dietary zinc intake (7.6 mg/d). In turn, Dempsey et al. [58] reported that marginal zinc deficiency in lactating mice reduces secretory capacity and alters milk composition. In our study, maternal total zinc intake (diet + supplementation) was positively correlated with its concentration in HM. What is more, in multivariable logistic regression, body fat mass, percentage of energy delivered from proteins, consumption frequency of meat, vegetables and legumes were revealed to be significant factors influencing zinc concentration in HM. Other studies have also showed a positive association between HM zinc and the intake of macronutrients or certain food groups, such as meat [23], fruit and rice [59] and total energy intake [60].

Considering that, in exclusively breastfed infants, HM is the only source of nutrition for the first few months of life, it is very important to have accurate data of its composition. Both analyzed minerals are essential for optimal infants’ growth and development. Iron deficiency in infancy may lead to poor psychomotor development, whereas zinc deficiency may result in a compromised immune system and stunned growth [34,61].

To our knowledge, this study is the first of its kind from Poland providing a comprehensive assessment of iron and zinc concentrations in HM in correlation with many maternal factors. The procedure of milk sample collection was cautiously planned and performed to minimize errors resulting from physiological factors and subsequently affecting HM composition. The additional strength of the present study is the assessment of maternal diet based on the combination of two methods—current and habitual intake. Furthermore, an advanced technique to assess maternal body composition was used. The present study also has several limitations. Firstly, convenience sampling and a modest number of participants. Secondly, to assess the daily milk volume, test weighting was used. According to some authors [27,62] in this method, milk intake is usually underestimated by approximately 1%–5% because of evaporative water loss from the infant between weighting. Taking it into consideration, in our study, the daily intake of investigated nutrients would be even lower. Finally, we did not assess iron and zinc status by evaluating plasma or serum levels of both mothers and babies, which may reveal if low dietary intake of minerals translates to maternal and infant’s plasma status.

## 5. Conclusions

Overall, the present study indicated that maternal diet influences iron and zinc concentrations in HM. Interestingly, a positive correlation between the concentration of both minerals in HM and women’s diet, was found only with their total dietary intake (diet + supplementation), indicating that maternal supplementation may be a positive factor for iron and zinc content in HM. Moreover, this study demonstrated that, among Polish infants at the age of 4–6 weeks, HM iron intakes were low, owing to low volumes of HM intake, despite HM iron concentration being in the normal range. In 15 samples, the concentration of zinc in HM was below the lower limit of quantification, hence the infant’s intake of zinc from HM was not calculated. Since the present study has the limitation of small sample size, further researches on a large scale, to investigate critical dietary factors that are associated with iron and zinc concentrations are warranted. Additionally, promotion of breastfeeding and thereby increasing the volume of produced HM may be a first crucial step towards improving minerals intake among infants. 

## Figures and Tables

**Table 1:** Work program of microwave digestion system.

Number	Time (Minutes)	Microwave Power (W)	Temperature (°C)
1	15	1800	0–180
2	10	1800	180
3	10	0	180–0

**Table 2:** Optimal measurement conditions of iron and zinc analysis by the ICP-MS method.

Parameter	Description
Spray Chamber	Scott, Quartz
Atomizer	Coaxial (Mainhardt)
Plasma torch	Quartz
Sampling cone	Nickel
The forming cone	Nickel
Frequency of the generator	40 MHz
Resolution of mass signals	(0.7 ± 0.05) AMU ^1^
Generator power	1600 W
Deflector voltage	−9 V
Voltage on the analog detector	−1800
Voltage on the pulse detector	950 V
Plasma gas flow	18.00 L/min
Auxiliary gas flow	1.2 L/min
The flow of atomizing gas	1.03 L/min
Stop time	50 ms
Number of sweeps	20
Number of repetitions	3–5
Solution dispensing speed	1 mL/min

^1^ Atomic mass unit.

**Table 3:** Characteristics of the study population.

Parameters	Mean ± SD	Median (Interquartile Ranges)
Age (years)	33.8 ± 4.6	32 (30.8–36)
Gestational age (weeks)	38 ± 1.2	39 (38–39)
Weight before pregnancy (kg)	62.2 ± 11.8	58 (53.4–70.6)
Weight gain during pregnancy (kg)	15.1 ± 4.7	14 (12–17)
Pre-pregnancy BMI (kg/m^2^)	22.6 ± 3.4	22.1 (19.6–24.2)
Current weight (kg)	65.5 ± 13.2	64.9 (54.5–74.5)
Current BMI (kg/m^2^)	23.6 ± 3.7	23.1 (20.4–26.3)
Rest metabolic rate (kcal)	1528.7 ± 72.1	1515 (1477.5–1578)
Fat free mass (%)	70.9 ± 9.0	70.2 (65.6–79.1)
Fat mass (%)	29.1 ± 9.0	29.7 (20.9–34.44)
Total body water (%)	50.9 ± 5.5	50.0 (46.2–55.5)
Extracellular body water (%)	46.4 ± 3.0	46.7 (45.4–48.0)
Intracellular body water (%)	53.6 ± 3.0	53.3 (52.0–54.6)
Body cell mass (kg)	24 ± 3.1	24.0 (22.2–26.9)
Proteins (kg)	8.8 ± 1.4	8.9 (8.3–9.7)
Minerals (kg)	3.7 ± 0.6	3.7 (3.4–4.0)
Muscles (kg)	19.8 ± 2.0	19.6 (18.5–21.3)
Infants’ birth weight (grams)	3548 ± 383	3530 (3225–3862.5)

**Table 4:** The composition of HM and estimated nutrients intake.

	Mean ± SD	Median (Interquartile Ranges)
**HM Composition**		
Energy (kcal/100 mL)	65.67 ± 10.73	65.5 (62–73.08)
Total protein (g/100 mL)	1.21 ± 0.23	1.17 (1.09–1.3)
True protein (g/100 mL)	0.96 ± 0.19	0.93 (0.8–1.01)
Fat (g/100 mL)	3.54 ± 1.04	3.55 (3.12–4.34)
Carbohydrates (g/100 mL)	6.94 ± 0.34	6.98 (6.77–7.18)
Iron (mg/L)	0.39 ± 0.2	0.33 (0.26–0.46)
Zinc (mg/L)	2.21 ± 0.37	2.12 (1.97–2.45)
Daily HM volume (ml)	492.5 ± 48	492.5 (466–528.5)
Number of daily feedings	10.3 ± 3.3	10 (8–11.25)
**Infants Intake of Energy and Nutrients ^1^**		
Energy (kcal/d)	324.55 ± 66.83	326.62 (284.78–363.35)
True protein (g/d)	4.74 ± 1.24	4.65 (3.96–5.18)
Fat (g/d)	17.54 ± 5.72	17.66 (15.35–21.1)
Carbohydrates (g/d)	34.15 ± 3.27	33.87 (31.79–36.71)
Iron (mg/d)	0.19 ± 0.1	0.16 (0.12–0.24)

^1^ Energy and nutrients intake from HM were calculated by multiplying the nutrient or energy content and mean daily milk intake.

**Table 5:** Correlations between maternal diet and supplementation and concentrations of iron and zinc in HM.

Energy and Nutrients in Maternal Diets	Mean ± SD	Median (Interquartile Ranges)	Spearman/Pearson Correlation Coefficient *r* ^1^	
			HM Iron Concentration	HM Zinc Concentration
Energy (kcal)	1831 ± 485.	1841 (1493–2104)	−0.201	−0.148
Protein (g)	78.6 ± 22.3	78.1 (61.8–92.4)	−0.011	0.12
Protein (% kcal)	17.5 ± 3.5	17.8 (14.7–19.9)	0.14	0.388
Fat (g)	63.8 ± 21	62.7 (50.4–73.9)	−0.184	−0.165
Fat (% kcal)	31 ± 5.9	30.1 (27.4–34.4)	0.045	−0.308
PUFA (g)	0.33 ± 0.49	0.08 (0.04–0.46)	−0.028	0.03
Carbohydrates (g)	255.1 ± 72.1	267.3 (190.3–296.7)	−0.183	0.009
Carbohydrates (% kcal)	51.5 ± 6.6	52.1 (48.1–55.7)	−0.149	−0.145
Sucrose (g)	49.6 ± 32.5	39.5 (24.3–61.4)	0.003	−0.167
Lactose (g)	13.2 ± 11.6	11.8 (5.3–20.5)	0.026	−0.167
Fiber (g)	21.3 ± 5.9	20.8 (16.8–25.4)	−0.023	−0.012
Sodium (mg)	2666 ± 874	2620 (2062–3160)	0.241	−0.001
Potassium (mg)	3023 ± 659	2924 (2516–3476)	−0.001	0.017
Calcium (mg)	746 ± 358	710 (517–914)	0.129	−0.113
Phosphorus (mg)	1331 ± 356	1288 (1042–1482)	−0.012	−0.112
Magnesium (mg)	317 ± 83	306 (267–336)	−0.089	−0.088
Iron (mg)	11.2 ± 2.8	10.9 (9.3–13)	−0.055	<0.001
Iron ^2^ (mg)	29.2 ± 20.3	17.5 (11.6–46.7)	0.258	0.452
Zinc (mg)	9.9 ± 1.8	9.6 (8.8–11)	0.104	0.001
Zinc ^2^ (mg)	13.6 ± 9	9.8 (9–12.3)	0.237	0.688 *
Iodine (µg)	106.3 ± 34.6	102.2 (86.3–127.2)	0.124	0.067
Vitamin A (µg)	1160.5 ± 579	1023.9 (825.8–1275.1)	−0.134	−0.4
Vitamin D (µg)	3 ± 2.4	1.9 (1.3–3.5)	0.081	0.366
Vitamin E (mg)	10.1 ± 4.2	9.3 (6.7–12.9)	0.129	−0.181
Vitamin B_1_ (mg)	1.2 ± 0.4	1.1 (1–1.3)	−0.199	−0.188
Vitamin B_2_ (mg)	1.7 ± 0.6	1.7 (1.3–2)	0.02	0.135
Vitamin PP (mg)	15.7 ± 6.1	14.6 (10.8–17.7)	−0.071	0.341
Vitamin B_6_ (mg)	1.8 ± 0.5	1.7 (1.4–2.1)	−0.074	0.13
Folic acid (mg)	300.3 ± 82.6	281.1 (239.2–371.3)	0.04	0.007
Vitamin B_12_ (µg)	3.7 ± 1.7	3.4 (2.3–4.7)	0.061	0.245
Vitamin C (mg)	125 ± 106.7	92 (63.6–130.8)	0.154	0.437

^1^ Spearman and Pearson Correlation Coefficient were used depending on variable distribution. ^2^ Diet + supplementation. * *p* < 0.05.

**Table 6:** Food intake frequencies and correlation with iron and zinc concentrations in HM.

Food Group	Median Values (Interquartile Ranges) of the Food Consumption Frequency ^1^	Spearman Correlation Coefficient *r*	
		HM Iron Concentration	HM Zinc Concentration
Grain based products (include 6 food items)	20 (17.5–22)	0.159	0.227
Milk and milk products (include 10 food items)	28 (13.5–33)	0.159	0.113
Eggs (include 1 food item)	3.5 (3–4)	0.400 *	−0.069
Meat (include 8 food items)	28 (23.75–31.25)	−0.01	0.12
Fish and seafood (include 3 food items)	28 (23.75–31.25)	−0.136	0.025
Vegetables and legumes (include 4 food items)	16 (10.75–16.25)	0.497 *	0.114
Fruits (include 5 food items)	18 (16–20)	0.012	0.408
Nuts and seeds (include 2 food items)	7 (5.75–8)	−0.105	0.23
Vegetable oils (include 6 food items)	10.5 (8–15)	0.165	−0.092
Sweets and snacks (include 8 food items)	22 (16.75–26)	0.169	0.443

^1^ The response options were arranged in five categories from ‘never’ (0 points), ‘less than once a week’ (1 point), ‘once or twice a week’ (2 points), ‘more than twice a week but not every day’ (3 points), to ‘every day’ (4 points). * *p* < 0.05.

**Table 7:** Results of multivariable linear regression analysis for prediction of HM iron concentration (Model A and Model C) and infants iron intake (Model B and D) ^1^.

	Estimate	Standard Error	*p*-Value
**Model A (Dependent Variable: HM Iron Concentration)**			
Supplemented iron (mg/d)	0.003	0.002	0.497
**Model B (Dependent Variable: Iron Infants’ Intake)**			
Total daily iron intake (mg/d)	0.002	0.0008	0.0198
**Model C (Dependent Variable: HM Iron Concentration)**			
Meat ^2^	0.018	0.008	0.046 *
Fish and seafood ^2^	−0.020	0.007	0.013 *
Vegetables and legumes ^2^	0.032	0.007	<0.001 *
Nuts and seeds	−0.029	0.013	0.037 *
Total daily iron intake (mg/d)	0.005	0.001	<0.001 *
**Model D (Dependent Variable: Iron Infants’ Intake)**			
Meat ^2^	0.009	0.003	0.030
Fish and seafood ^2^	−0.011	0.004	0.005
Vegetables and legumes ^2^	0.015	0.003	<0.001 *
Nuts and seeds ^2^	−0.014	0.006	0.039 *
Total daily iron intake (mg/d)	0.002	0.001	<0.001

^1^ Iron concentration in HM for models A and C, while infants’ iron intake in HM for models B and D were used as dependent variables, respectively. For models A and B, mother’s age, actual body mass, body fat mass, percentage of energy delivered from macronutrients (protein, fat, carbohydrates), dietary, supplemented and total iron intakes, were considered as explanatory variables. For models C and D mother’s age, actual body mass, body fat mass, consumption frequency of all selected food groups (meat, fish and seafood, vegetables and legumes, nuts and seeds), maternal dietary, supplemented and total iron intakes were evaluated as explanatory variables. ^2^ Results from FFQ, consumption frequency. Intercepts were not reported. * *p* < 0.05.

**Table 8:** Results of multivariable logistic regression analysis for the prediction of HM zinc concentration higher than 90th percentile ^1^.

	Odds Ratio	95% Confidence Interval	*p*-Value
**Model E (Dependent Variable: HM Zinc Concentration)**			
Body fat mass (%)	0.89	0.63–1.11	0.357
Percentage of energy delivered from protein (%)	2.00	1.02–7.81	0.141
Total zinc intake (mg/d)	1.18	1.04–1.52	0.056
**Model F (Dependent Variable: HM Zinc Concentration)**			
Meat^2^	1.382	0.96–2.69	0.161
Vegetables and legumes^2^	1.279	0.87–2.76	0.340
Total zinc intake (mg/d)	1.227	1.06–1.65	0.034

^1^ Zinc concentration in HM higher than 90th percentile was used as dependent variable in both models. For model E mother’s age, actual body mass, body fat mass, percentage of energy delivered from all macronutrients (protein, fat, carbohydrates) dietary, supplemented and total zinc intakes were considered explanatory variables. For model F mother’s age, actual body mass, body fat concentration, consumption frequency of all investigated food groups, supplemented and total zinc intakes, were evaluated as explanatory variables. Odds ratios were computed for 1-unit increase of each variable. ^2^ Results from FFQ, consumption frequency.

## Data Availability

The data presented in this study are available on request from the corresponding author. The data are not publicly available due to information that could compromise the privacy of research participants.

## References

[1] WHO (2013). Global Strategy for Infant and Young Child Feeding.

[2] Picciano M.F. (2001). Nutrient Composition of Human Milk. Pediatr. Clin. North Am..

[3] Eussen S., Alles M., Uijterschout L., Brus F., van der Horst-Graat J. (2015). Iron intake and status of children aged 6–36 months in Europe: A systematic review. Ann. Nutr. Metab..

[4] Dror D.K., Allen L.H. (2018). Overview of nutrients in human milk. Adv. Nutr..

[5] Hutton E.K., Hassan E.S. (2007). Late vs early clamping of the umbilical cord in full-term neonates: Systematic review and meta-analysis of controlled trials. J. Am. Med. Assoc..

[6] Institute of Medicine (1991). Nutrition during Lactation.

[7] Chaparro C.M. (2008). Setting the stage for child health and development: Prevention of iron deficiency in early infancy. J. Nutr..

[8] Mastroeni S.S., Okada I.A., Rondo P.H., Duran M.C., Paiva A.A., Neto J.M. (2006). Concentrations of Fe, K, Na, Ca, P, Zn and Mg in maternal colostrum and mature milk. J. Trop. Pediatr..

[9] Dorea J.G. (2000). Iron and copper in human milk. Nutrition.

[10] Wasowicz W., Gromadzinska J., Szram K., Rydzynski K., Cieslak J., Pietrzak Z. (2001). Selenium, zinc, and copper concentrations in the blood and milk of lactating women. Biol. Trace Elem. Res..

[11] Jarosz M., Rychlik E., Stoś K., Charzewska J. (2020). Dietary Reference Values for the Polish Population and their Application.

[12] Ackland L., Michalczyk A. (2016). Zinc and infant nutrition. Arch. Biochem. Biophys..

[13] Black R.E., Fischer Walker C. (2012). Role of zinc in child health and survival. Nestle Nutr. Inst. Workshop.

[14] EFSA NDA Panel (2014). Scientific Opinion on Dietary Reference Values for Zinc. EFSA J..

[15] Brown K.H., Engle-Stone R., Krebs N.F., Peerson J.M. (2009). Dietary intervention strategies to enhance zinc nutrition: Promotion and support of breastfeeding for infants and young children. Food Nutr. Bull..

[16] Wilson R.L., Grieger J.A., Bianco-Miotto T., Roberts C.T. (2016). Association between Maternal Zinc Status, Dietary Zinc Intake and Pregnancy Complications: A Systematic Review. Nutrients.

[17] Farias P.M., Marcelino G., Santana L.F., de Almeida E.B., Guimarães R., Pott A., Hiane P.A., Freitas K.C. (2020). Minerals in Pregnancy and Their Impact on Child Growth and Development. Molecules.

[18] Krebs N.F., Westcott J. (2002). Zinc and breastfed infants: If and when is there a risk of deficiency?. Adv. Exp. Med. Biol..

[19] Maguire J.L., Salehi L., Birken C.S., Carsley S., Mamdani M., Thorpe K.E. (2013). Association between total duration of breastfeeding and iron deficiency. Pediatrics.

[20] Dumrongwongsiri O., Suthutvoravut U., Chatvutinun S., Phoonlabdacha P., Sangcakul A., Siripinyanond A. (2015). Maternal zinc status is associated with breast milk zinc concentration and zinc status in breastfed infants aged 4–6 months. Asia Pac. J. Clin Nutr..

[21] Clark K.M., Li M., Zhu B., Liang F., Shao J., Zhang Y. (2017). Breastfeeding, mixed, or formula feeding at 9 months of age and the prevalence of iron deficiency and iron deficiency anemia in two cohorts of infants in China. J. Ped..

[22] Lönnerdal B. (2000). Regulation of mineral and trace elements in human milk: Exogenous and endogenous factors. Nutr. Rev..

[23] Choi Y.K., Kim J.M., Lee J.E., Cho M.S., Kang B.S., Choi H., Kim Y. (2016). Association of maternal diet with zinc, copper, and iron concentrations in transitional human milk produced by Korean mothers. Clin. Nutr. Res..

[24] Hannan M.A., Faraji B., Tanguma J., Longoria N., Rodriguez R.C. (2009). Maternal milk concentration of zinc, iron, selenium, and iodine and its relationship to dietary intakes. Biol. Trace Elem. Res..

[25] Yalçin S.S., Baykan A., Yurdakök K., Yalçin S., Gücüş A.I. (2009). The factors that affect milk-to-serum ratio for iron during early lactation. J. Pediatr. Hematol. Oncol..

[26] Mahdavi R., Nikniaz L., Gayemmagami S.J. (2010). Association between zinc, copper, and iron concentrations in breast milk and growth of healthy infants in Tabriz, Iran. Biol. Trace Elem. Res..

[27] Silvestre M.D., Lagarda M.J., Farre R., Martinez-Costa C., Brines J., Molina A., Clemente G. (2000). A study of factors that may influence the determination of copper, iron, and zinc in human milk during sampling and in sample individuals. Biol. Trace Elem. Res..

[28] Winiarska-Mieczan A. (2014). Cadmium, Lead, Copper and Zinc in Breast Milk in Poland. Biol. Trace. Elem. Res..

[29] Woolridge M.W., Butte N., Dewey K.G., Ferris A.M., Garza C., Keller E.P., Jensen R.G., Neville M.C. (1985). Methods for the measurement of milk volume intake of the breastfed infant. Human Lactation: Milk Components and Methodologies.

[30] Szponar L., Wolnicka K., Rychlik E. (2011). Albums of Photographs of Food Products and Dishes.

[31] World Health Organization (WHO) Fourth WHO Coordinated Survey of Human Milk for Persistent Organic Pollutants in Cooperation with UNEP. Guidelines for Developing a National Protocol. http://www.who.int/foodsafety/chem/POPprotocol.pdf.

[32] Euro WHO. https://www.euro.who.int/en/health-topics/disease-prevention/nutrition/a-healthy-lifestyle/body-mass-index-bmi.

[33] Heyvard V.H., Stolarczyk L.M. (1996). Applied Body Composition Assessment.

[34] Bzikowska-Jura A., Czerwonogrodzka-Senczyna A., Olędzka G., Szostak-Węgierek D., Weker H., Wesołowska A. (2018). Maternal Nutrition and Body Composition During Breastfeeding: Association with Human Milk Composition. Nutrients.

[35] Bzikowska-Jura A., Sobieraj P., Szostak-Węgierek D., Wesołowska A. (2020). Impact of Infant and Maternal Factors on Energy and Macronutrient Composition of Human Milk. Nutrients.

[36] Domellöf M., Lönnerdal B., Dewey K.G., Cohen R.J. (2004). Hernell, Iron, zinc, and copper concentrations in breast milk are independent of maternal mineral status. Am. J. Clin. Nutr..

[37] Gibson R.S., Rahmannia S., Diana A., Leong C., Haszard J.J., Hampel D., Reid M., Erhardt J., Suryanto A.H., Sofiah W.N. (2020). Association of maternal diet, micronutrient status, and milk volume with milk micronutrient concentrations in Indonesian mothers at 2 and 5 months postpartum. Am. J. Clin. Nutr..

[38] Dumrongwongsiri O., Chongviriyaphan N., Chatvutinun S., Phoonlabdacha P., Sangcakul A., Siripinyanond A., Suthutvoravut U. (2020). Dietary Intake and Milk Micronutrient Levels in Lactating Women with Full and Partial Breastfeeding. Matern. Child Health J..

[39] Nakamori M., Ninh N.X., Isomura H., Yoshiike N., Hien V.T.T., Nhug B.T., Van Nhien N., Nakano T., Khan N.C., Yamamoto S. (2009). Nutritional status of lactating mothers and their breast milk concentration of iron, zinc and copper in rural Vietnam. J. Nutr. Sci. Vitaminol..

[40] Aumeistere L., Ciproviča I., Zavadska D., Bavrins K., Borisova A. (2018). Zinc Content in Breast Milk and Its Association with Maternal Diet. Nutrients.

[41] Doneray H., Olcaysu E., Yildirim A., Ozden A. (2017). The effect of the zinc concentration in breast milk on neonatal weight gain. J. Trace Elem. Med. Biol..

[42] Yoshinaga J., Li J.Z., Suzuki T., Karita K., Abe M., Fujii H., Mishina J., Morita M. (1991). Trace elements in human transitory milk. Variation caused by biological attributes of mother and infant. Biol. Trace Elem. Res..

[43] Krebs N.F., Hambidge M., Jacobs M.A., Mylet S. (1985). Zinc in human milk: Diurnal and within-feeding patterns. J. Pediatr. Gastroenterol. Nutr..

[44] Celada A., Busset R., Gutierrez J., Herreros V. (1982). No correlation between iron concentration in breast milk and maternal iron stores. Helv. Paediatr. Acta.

[45] Fransson G.B., Agarwal K.N., Gebre M.M., Hambraeus L. (1985). Increased breast milk iron in severe maternal anemia: Physiological “trapping” or leakage?. Acta Paediatr. Scand..

[46] Tamura T., Yoshimura Y., Arakawa T. (1980). Human milk folate status in lactating mothers and their infants. Am. J. Clin. Nutr..

[47] Mello-Neto J., Carvalho Rondó P.H., Oshiiwa M., Morgano M.A., Zago Zacari C., Lima dos Santos M. (2013). Iron Supplementation in Pregnancy and Breastfeeding and Iron, Copper and Zinc Status of Lactating Women from a Human Milk Bank. J. Trop. Ped..

[48] O’Brien K.O., Zavaleta N., Caulfield L.E., Wen J., Abrams S.A. (2000). Prenatal iron supplements impair zinc absorption in pregnant Peruvian women. J. Nutr..

[49] Neville M.C., Keller R., Seacat J., Lutes V., Neifert M., Casey C., Allen J., Archer P. (1988). Studies in human lactation: Milk volumes in lactating women during the onset of lactation and full lactation. Am. J. Clin. Nutr..

[50] Butte N.F., Lopez-Alarcon M.G., Garza C. (2002). Nutrient Adequacy of Exclusive Breastfeeding for the Term Infant During the First Six Months of Life.

[51] Fomon S.J. (1993). Nutrition of Normal Infants.

[52] Samuel T.M., Thomas T., Thankachan P., Bhat S., Virtanen S.M., Kurpad A.V. (2014). Breast milk zinc transfer and early post-natal growth among urban South Indian term infants using measures of breast milk volume and breast milk zinc concentrations. Matern. Child Nutr..

[53] Simmer I., Ahmed S., Carlsson L., Thompson R.P.H. (1990). Breast milk zinc and copper concentrations in Bangladesh. Br. J. Nutr..

[54] Karra M.V., Kirksey A., Galal O., Bassily N.S., Harrison G.G., Jerome N.W. (1988). Zinc, calcium, and magnesium concentrations in milk from American and Egyptian women throughout the first 6 mo of lactation. Am. J. Clin. Nutr..

[55] Arnaud J., Prual A., Preziosi P., Cherouvrier F., Favier A., Galan P., Hercberg S. (1993). Effect of iron supplementation during pregnancy on trace element (Cu, Se, Zn) concentrations in serum and breast milk from Nigerian women. Ann. Nutr. Metab..

[56] Krebs N.F., Reidinger C.J., Hartley S., Robertson A.D., Hambidge K.M. (1995). Zinc supplementation during lactation: Effects on maternal status and milk zinc concentrations. Am. J. Clin Nutr..

[57] Sian L., Krebs N.F., Westcott J.E., Fengliang L., Tong L., Miller L.V. (2002). Zinc homeostasis during lactation with a low zinc intake. Am. J. Clin. Nutr..

[58] Leotsinidis M., Alexopoulos A., Kostopoulou-Farri E. (2005). Toxic and essential trace elements in human milk from Greek lactating women: Association with dietary habits and other factors. Chemosphere.

[59] Vuori E., Makinen S.M., Kara R., Kuitunen P. (1980). The effects of the dietary intakes of copper, iron, manganese, and zinc on the trace element content of human milk. Am. J. Clin. Nutr..

[60] Dempsey C., McCormick N.H., Croxford T.P., Seo Y.A., Grider A., Kelleher S.L. (2012). Marginal maternal zinc deficiency in lactating mice reduces secretory capacity and alters milk composition. J. Nutr..

[61] Petry N., Olofin I., Boy E., Donahue Angel M., Rohner F. (2016). The Effect of Low Dose Iron and Zinc Intake on Child Micronutrient Status and Development during the First 1000 Days of Life: A Systematic Review and Meta-Analysis. Nutrients.

[62] Brown K.H., Black R.E., Robertson A.D., Akhtar N.A., Ahmed G., Becker S. (1982). Clinical and field studies of human lactation: Methodological considerations. Am. J. Clin. Nutr..

